# Super donor assessment tool for oral microbiome transplantation

**DOI:** 10.1186/s12866-025-04630-z

**Published:** 2025-12-23

**Authors:** Sonia Nath, Murthy Mittinty, Peter Zilm, Pedro Henrique Ribeiro Santiago, Don Kevin Hashan Ketagoda, Lisa Jamieson, Laura Weyrich

**Affiliations:** 1 Adelaide Dental School, The University of Adelaide, Adelaide, SA 5000 Australia; 2 School of Biological Sciences, University of Adelaide, Adelaide, SA Australia; 3 College of Medicine and Public Health, Flinders University, Adelaide, SA Australia; 4 Department of Anthropology, The Pennsylvania State University, University Park, PA 16802 USA

**Keywords:** Analytical hierarchy process, Decision support model, Decision making, Microbiome, Microbiota, Oral health

## Abstract

**Aims:**

Oral microbiome transplantation (OMT) involves transferring microbiota from donor to recipient. However, selecting suitable donors remains challenging due to a lack of standardised guidelines. This study developed a novel super donor assessment tool (SDAT) combining a multi-criteria decision-making (MCDM) process and an analytical hierarchical process (AHP) to identify OMT “super donors” for dental caries prevention.

**Methods:**

This cross-sectional study used four sequential screening phases with data from 93 healthy participants, capturing socio-demographics, lifestyle, dietary and oral health behaviours. The SDAT employed MCDM, AHP, combining criteria with normalised and weighted ranks to establish the top 10 donors for three models: “Optimal donor” (Model 1), “Ideal donor” (Model 2), and “Sub-optimal donor” (Model 3). Donor plaque samples underwent 16S ribosomal RNA amplicon sequencing for microbial profiling, examining alpha and betadiversity, differential abundance, and network analysis.

**Results:**

Alpha diversity analysis showed significant differences among groups (Kruskal-Wallis *p* < 0.001), with Model 1 showing the lowest diversity and Model 3 the highest. Beta diversity analysis using Permutational Multivariate Analysis of Variance revealed significant differences in microbial community composition (R² = 0.19, *p* = 0.001). Differential abundance analysis (False Discovery Rate < 0.05, controlling for age and sex) identified health-associated genera (*Neisseria*,* Lautropia*,* Streptococcus*,* Veillonella*) in Model 1, whereas Model 3 showed higher levels of disease-associated taxa (*Treponema*,* Capnocytophaga*). Network analysis revealed that Model 1 was organised around *Actinomyces* and *Prevotella*, Model 2 around *Rothia* and *Haemophilus*, and Model 3 was dominated by pathogenic taxa.

**Conclusion:**

SDAT provides a systematic, transparent framework for super-donor selection, ensuring precision and reproducibility in donor rankings. The scoring system standardises the donor selection process, the effectiveness of donor screening, and reduces the risk of adverse events for OMT.

**Supplementary Information:**

The online version contains supplementary material available at 10.1186/s12866-025-04630-z.

## Introduction

Oral microbiome transplantation (OMT) is emerging as a promising therapeutic strategy for managing common oral diseases such as dental caries and periodontal disease by restoring microbial balance in the oral cavity through the transfer of health-associated microbiota from a healthy donor to the recipient [1, 2]. This innovative intervention builds upon the established principles of faecal microbiome transplantation (FMT), which has shown considerable success in treating gastrointestinal conditions [3], while adapting the methodology specifically for the unique environment of the oral cavity [4]. The goal is to establish a new microbial community that can effectively prevent or treat oral diseases by shifting the recipient’s microbial profile toward one associated with health rather than disease. The nuanced understanding of the oral microbiome’s role in different disease states underscores the importance of tailored therapeutic strategies rather than a one-size-fits-all approach to microbial transplantation. This fundamental difference necessitates disease-specific approaches to OMT, especially for caries prevention. A multitude of factors can influence a donor’s oral microbiome, as it is a complex and dynamic system consisting of bacteria, viruses, and fungi, and the diversity of the healthy oral microbiome can vary significantly between individuals, and specific microbial communities may be more health-associated than others [5, 6]. A combination of host, environmental, behavioural and nutritional factors could help identify the presence and abundance of beneficial bacteria associated with health [7–9].

Central to the success of OMT is the identification and selection of “super donors” whose oral microbiota possess specific characteristics that would result in successful transplantation. The term “super donor” originated in FMT research to describe individuals whose microbiota confer significantly higher rates of successful outcomes compared to other donors [10]. Some individuals present a paradox in oral health, remaining caries-free despite exhibiting lifestyle factors such as high sugar intake, infrequent tooth brushing, or irregular dental visits that commonly predispose to dental caries. This resilience may be driven by the unique traits of their oral microbiome, which demonstrates an exceptional capacity to resist cariogenic challenges and maintain oral health. These “super” individuals may harbour microbial communities characterised by low levels of pathogenic cariogenic species such as *Streptococcus mutans (S. mutans)*, while maintaining a predominance of health-associated taxa that buffer acids and suppress the pathobionts. Identifying the donors for FMT is currently a *post hoc* analysis [11]. In the context of OMT, identifying these “super-donors” requires rigorous screening and assessment of multiple factors that collectively contribute to a health-promoting oral microbiome [9]. While initial animal and clinical OMT studies have provided proof-of-concept data, robust evidence specifically evaluating efficacy for dental caries in adult human populations remains limited. In an initial proposed protocol for OMT, the authors suggested a direct transfer of oral bacteria from a healthy donor to a recipient, such as a spouse or partner [12]. The first OMT study used a dog model of naturally occurring periodontitis [4], with a single donor to a single recipient. This was followed by using OMT in animal experiments for mucositis [13] and intraoral halitosis [14]. The first-ever clinical OMT was performed in the mother-to-child transfer for oral mucositis and primarily reflects microbiome establishment in infancy, a period marked by rapid and profound microbial shifts that differ fundamentally from the more stable adult oral ecosystem [15]. However, recent studies in animal models and exploratory human research have demonstrated that targeted manipulation of the oral microbiome is feasible and can shift recipients’ microbial profiles towards health. Ongoing and upcoming human trials continue to evaluate OMT safety and efficacy, underscoring the translational potential of this approach. The development of objective super donor assessment tools and tailored transplantation protocols fosters the clinical translation of OMT for caries and oral disease prevention in adults.

Currently, there are no guidelines for choosing suitable oral microbiome donors. In all the OMT studies, the criteria for donor selection were either not explored or chosen at random [4, 13–15]. The development of an OMT super donor assessment tool (SDAT) would be beneficial in objectively evaluating potential donors. The tool was based on a combination of multiple-phase screening, assessed using normalised, weighted ranks and multiple criteria decision-making (MCDM) processes within an analytical hierarchical process (AHP) [16–18]. Such a tool would provide a systematic, structured, and transparent framework for super donor selection, allowing for precise and reproducible calculations of super donor ranks rather than subjective assessments. Therefore, the goal of this study was to develop and test the super donor assessment tool for donor selection for the caries prevention model.

## Methods

### Study design and participant screening process

This study was conducted from June 2021 to July 2022 at the Adelaide Dental School, University of Adelaide. The study was approved by the University of Adelaide Human Research Ethics Committee (H-2020-34609) and conducted in accordance with the Declaration of Helsinki for Medical Research. After explaining the purpose of the study and the expected outcome, written informed consent was obtained from each participant. The detailed protocol has been published previously [2].

**Fig. 1:**
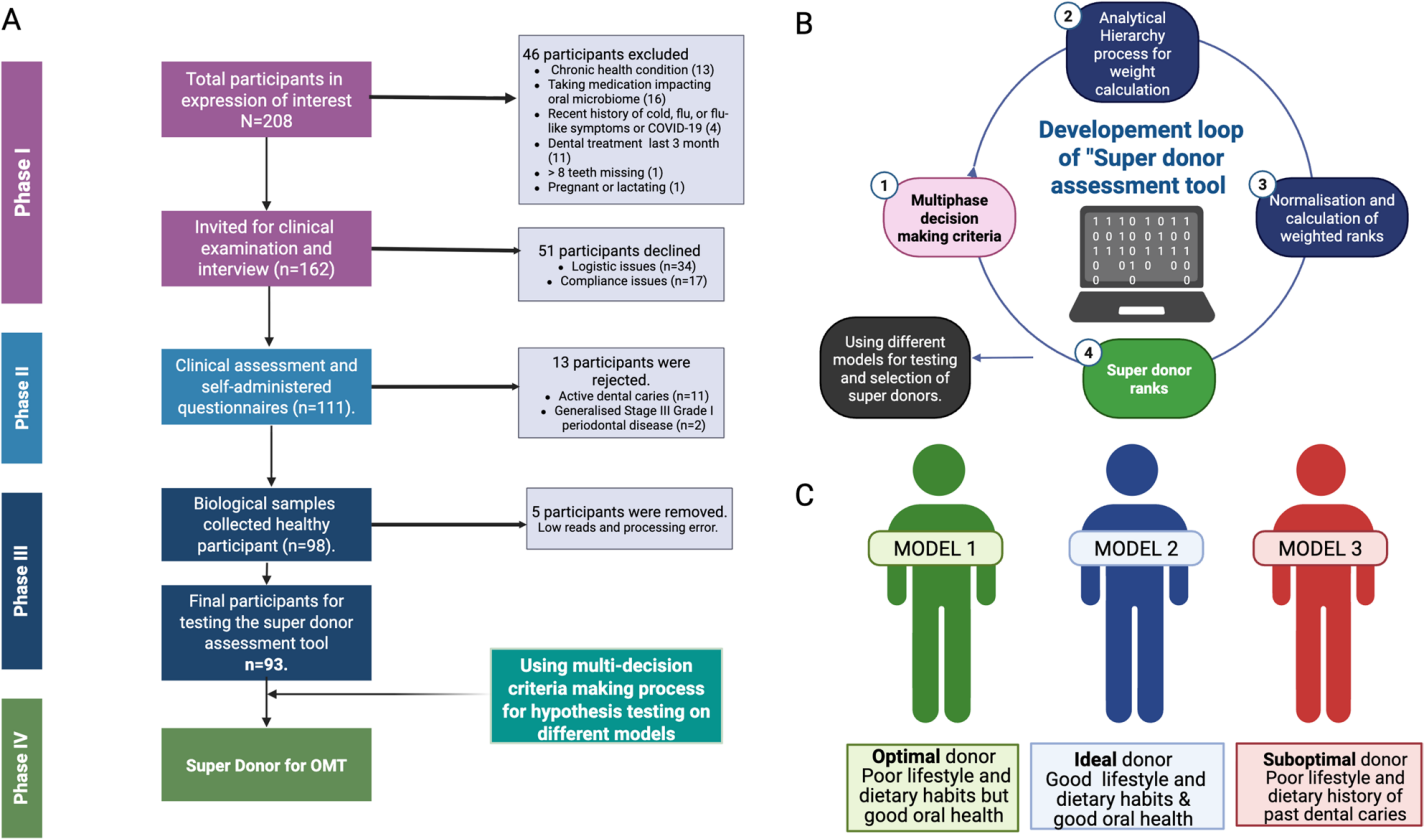
Study design and development of the super donor assessment tool for oral microbiota transplantation (OMT). **A** Flow diagram outlining participant recruitment and screening across four phases. In phase I, 208 participants showed interest, and in phase II, 111 underwent oral assessments, and participants with dental caries, periodontal disease, or chronic systemic conditions were excluded. The data, supragingival plaque, and stimulated saliva samples were collected from 98 healthy individuals in phase III. The super donor screening tool was applied based on weighted and normalised ranks for each criterion, and super donor rank was generated for 93 participants for Phase IV. The top 10 participants were selected from each model for further analysis. **B** Schematic of the super donor assessment tool development process. The tool was constructed using a multiphase decision-making framework, analytic hierarchy process (AHP) for weight calculation, normalisation and calculation of weighted ranks, and application of different models to generate super donor rankings. **C** Illustration of the three donor selection models used for hypothesis testing: Model 1 (*“Optimal donor”*) prioritises good oral health despite poor lifestyle and dietary habits; Model 2 (*“Ideal donor”)* represents individuals with both good lifestyle/dietary habits and good oral health; Model 3 (*“Suboptimal donor”*) includes those with poor lifestyle and dietary history and past dental caries

The participant selection process consisted of a four-stage screening process (Fig. 1A). Phase one involved pre-screening, where 208 participants were invited to participate in the study and complete a brief, self-administered questionnaire. Participants with dental caries, periodontal disease, or chronic systemic conditions were excluded [2]. Phase two consisted of the oral examination. Two registered dental practitioners (SN and KK) conducted oral assessments of 111 study participants, including a comprehensive examination of soft tissues, periodontal health, and dental health. Participants were included if bleeding on probing at all sites was ≤ 20% after 15 s of probing, the periodontal probing depth was ≤ 3 mm, and the clinical attachment levels were ≤ 4 mm to ensure the absence of oral disease. The Decayed, Missing, and Filled Teeth (DMFT) index was used to assess dental caries [19], and the cumulative “Missing” (M) and “Filled” (F) scores were used to evaluate past caries experience. Thirteen participants with either dental caries or periodontal disease were excluded. Phase three consisted of data and sample collection. The biological samples, including dental plaque and stimulated whole saliva were collected and 93 participants were included [9, 20]. All the participants were asked to complete two self-administered questionnaires. The first questionnaire captured information on sociodemographic and lifestyle factors, such as physical activity and smoking, dental behaviour, including tooth brushing and flossing, the last dental visit, and fluoride usage [2]. The second questionnaire used was the Dietary Questionnaire for Epidemiological Studies (DQES v3.2) [2]. The detailed covariate categorisation and sample collection protocol are described in Supplementary Text 1.1.

### DNA extractions, amplification, and sequencing

All DNA extractions from dental plaque were performed using the Qiagen DNeasy PowerSoil Pro kits (Qiagen, Hilden, Germany) in a controlled laboratory environment at the University of Adelaide, according to the manufacturer’s recommendations. For every set of 12 samples, two extraction blanks were included and processed alongside [21]. The extracted DNA was then shipped to the microARCH laboratory at The Pennsylvania State University for further processing. A unique barcoded reverse primer was used for each sample to amplify the V4 region of the bacterial 16 S rRNA, as previously described [22]. Non-template controls (NTC) were also processed alongside each amplification. The amplified, barcoded DNA was quantified using the Invitrogen Qubit dsDNA BR assay (Life Technologies). The samples were pooled together at equal relative concentrations, purified using 1.1X Axygen Axyprep Mag TM PCR clean-up beads, and then quantified using D1000 reagents on a Tapestation (Agilent, Santa Clara, CA, USA). Sequencing was performed on an Illumina MiSeq 2300 platform at the Genomic Core Facility, The Pennsylvania State University.

### Strategy for super donor assessment tool

The super donor ranking process involved four sequential computational steps (Fig. 1B). First, a multicriteria decision-making approach (MCDM) was used to select the variables [18]. The first step was to identify the key criteria for selecting super donors. Based on the literature, 12 dimensions were used, including lifestyle, behavioural, and nutritional factors [9, 20]. Each of these factors was assigned a specific weight in the assessment tool to reflect its relative importance in defining a super donor (Supplementary Text 1.2). These variables included the final pH difference after the glucose test, salivary flow rate, the number of missing teeth, and the number of restored or filled teeth, which were used as markers of dental health status. Fluoride application, specifically whether a fluoride varnish had been applied, was used as an indicator of preventive dental care. Sugar consumption and dietary fibre consumption were assessed to evaluate nutritional habits. Smoking status, the timing of the last dental visit for treatment, brushing frequency, and physical activity were included to capture lifestyle factors influencing oral health. Finally, the bacterial composition of the oral microbiome was evaluated by quantifying the abundance of key species: *S. mutans*, *Fusobacterium nucleatum* (*F. nucleatum*), *Porphyromonas gingivalis* (*P. gingivalis*), *Treponema denticola* (*T. denticola*), and *Tannerella forsythia* (*T. forsythia*).

A relative abundance table was generated using the “*qiime taxa barplot*” function to identify the abundance count of the following species: *S. mutans*, *F. nucleatum*, *P. gingivalis*, *T. denticola*, and *T. forsythia*. To determine the species identification of *F. nucleatum*, the Basic Local Alignment Search Tool nucleotide algorithm (BLASTN) was used. BLASTN is a tool that compares nucleotide sequences, such as DNA, to reference databases to find regions of similarity and help identify organisms. The *Fusobacterium* representative sequence was searched against the NCBI 16 S Microbial database using BLASTN [23]. The output was sorted based on the e-value, and there were five amplicon sequence variants (ASVs) of *Fusobacterium*, with only one taxon matching at 96% similarity [23]. The output was sorted based on the e-value, and there were five ASVs of *Fusobacterium*, with only one taxon matching at 96% similarity. Assessing super donors for these pathobiont species was crucial to safeguard against harm during OMT.

### Models for hypothesis testing

We developed three models to guide hypothesis testing for the dental caries prevention model, each based on different weighting factors that could influence the oral microbiome (Fig. 1C). For every model, the decision-maker (SN) assigned specific ranks to these factors as part of a structured decision-making process (Supplementary Table 1).

#### Model 1 “The optimal donor”

These optimal donors are individuals with no history of dental caries, despite unfavourable lifestyle and environmental factors. They would have high salivary pH after a glucose test, an optimal saliva flow rate (1.5–2.0 mL/min), and no missing or restored teeth. Additionally, they did not visit the dentist in the last 2 years or more, avoided or had minimal use of fluoride products, and brushed once a day. They do not exercise regularly (minimal activity), consume a diet high in sugar (> 100 g/d) and low in fibre (< 20 g/d), but do not smoke. Additionally, plaque samples from these donors were evaluated for the relative abundance of key oral pathogens, including *S. mutans*, *F. nucleatum*, *P. gingivalis*, *T. denticola*, and *T. forsythia*. Donors were ranked lower if any of these pathogenic species were in higher abundance.

#### Model 2 “Ideal donor”

The second model focused on the characteristics of an ideal donor, emphasising proactive oral health maintenance. These donors also have no history of caries, high saliva flow rates (1.5–2.0 mL/min), and high saliva pH after a glucose challenge. They maintain good oral hygiene by brushing at least twice daily, visiting the dentist every six months, and receiving regular professional fluoride treatments. Their lifestyle habits are generally healthy, including a diet low in sugar and high in fibre, along with regular exercise. As in Model 1, donors are ranked lower if they exhibit higher levels of the five specified oral pathogens.

#### Model 3 “Suboptimal donor”

In the third model, all variables from *“ideal donors”* are reversed to represent donors with characteristics least suitable for OMT super donor candidates. These donors have a history of restoration (> 4) and missing teeth (> 2), low saliva flow rate (< 1.4 ml/min), low saliva pH after glucose challenge, a sugar-rich diet (> 100 g/d), and fibre intake can be less than 20 g/day. Dental visits were more than two years ago, and they were current smokers. Brushing frequency was once daily, and there was no minimum requirement for physical activity. The abundance of oral pathogens in this group was higher, reflecting a less healthy microbiome profile, providing a contrast for comparison with the *“optimal”* and *“ideal donor”* models.

### Bioinformatics

QIIME version software (v.2022.11) [24] was used for pre-processing the data. The R (version 4.4.2) package *decontam* [25], was used to detect and remove microbes from three types of negative controls (extraction blank control, no template control, and curette wash) using a two-step process. The data preprocessing has been described previously [9, 20] and is detailed in Supplementary Text 2. After this step, a *phyloseq* object was created.

To evaluate and compare the three donor selection models (*“Optimal donor*”, *“Ideal donor”* and *“Sub-optimal donor*”), alpha and beta diversity metrics of the oral microbiome were calculated for each group with data rarefied to a consistent sampling depth to ensure comparability across samples. Alpha diversity was assessed using the “Observed” (number of observed taxa) and “Shannon” (both richness and evenness) indices. Violin plots were generated to visualise the distribution of alpha diversity within each model group, and statistical comparisons were performed using the Wilcoxon rank-sum test. Beta diversity was analysed using non-metric multidimensional scaling (NMDS) based on Aitchison distance to visualise differences in overall community composition between the three models. Permutational multivariate analysis of variance (PERMANOVA) was used to test for model differences in beta diversity, using Aitchison’s distance matrix with 999 permutations in the *vegan* package [26]. Compositional bar plots were created to display the relative abundance of the most prevalent microbial genera for each donor model. The data were aggregated at the genus level, focusing on the 12 most abundant genera and were visualised using the *MicroViz* package [27].

Core microbiome analysis identified genera present in at least 80% of samples (with a minimum abundance of five counts) within each model group. The unique and shared core genera among the three models were identified, and a Venn diagram was constructed to illustrate these relationships. Differential abundance analysis was performed at the genus level using MaAsLin2 to identify taxa that were significantly different from *Ideal (Model 2)*, which served as the reference group [28]. Age and sex were included as covariates to control for potential confounding effects. The count data was transformed into proportions using base Log2 linear regression with total sum scaling (TSS), and a forest plot was constructed for visualisation. Network analysis was performed with the *NetCoMi* for all three models at the genus level [29]. The analysis employed Pearson’s correlation after CLR normalisation and zero handling, with multiplicative imputations and filtering for features with fewer than 500 reads. The associations were measured using the Fruchterman-Reingold algorithm and greedy optimisation for network creation [30]. The *patchwork* package was used to arrange plots into a single figure [31].

## Results

### Developing the super donor assessment tool

For identifying a super donor, MCDM was used, which refers to screening, prioritising, ranking or selecting the variables (12 dimensions) from a large set of usually conflicting criteria [18]. In this paper, we used rank ordering weighting methods that take into account decision makers’ information about rank ordering of criteria weights, from which approximations for criteria weights are calculated by using the formula given in Eq. 2. The MCDM considered in this manuscript is described as follows:

Let $\:{V}_{1},{V}_{2},{V}_{3},\dots\:,{V}_{m}$ be various (*m*) aspects of interest that are observed in the study. Now, say we are interested in reducing the dimension of these multiple criteria into a single index value, which allows us to make decisions. Some common methods to use are principal component analysis, factor analysis or linear discriminant analysis. Like these methods in MCDM too we created weights, but these weights were created based on two criteria: benefit and cost. The benefit criterion is used to reflect the idea that features with higher values are always better. In the cost criterion, the features with lower values are always better. Under both these criteria we have a weight vector $\:w=[{w}_{1},{w}_{2},\dots\:,{w}_{k}]$ such that $\:{w}_{1}+{w}_{2}+\dots\:+{w}_{k}=1$ and $\:{w}_{k}>0.$ In this manuscript, we use the benefit criterion, which can be described as follows. Assume that $\:{x}_{ij}$ denote the observed value on each of the variable (*j* = 1,2,…,*m*) (*i=*1,2,…*n*) in the study. According to the benefit criterion, the observed data matrix is normalised using the following Eq. 1, such that the values of all variables lie in between 0 and 1.

1 $$\:{c}_{ij}=\frac{{x}_{ij}-\mathrm{m}\mathrm{i}\mathrm{n}\left({x}_{ij}\right)}{\mathrm{max}\left({x}_{ij}\right)-\mathrm{min}\left({x}_{ij}\right)}$$

For creating weights, one can use equal weight criteria (where every variable is given the same importance) or can create a weight using rank sum method or rank exponent method. However, we used user-specified weights, as follows $\:w=(0.05,\:0.2,\:0.1,\:0.05,\:0.05,\:0.05,\:0.20,\:0.10,\:0.05,\:0.05,\:0.10)$: A simple and most often used multi-criteria decision technique is the *Simple Additive Weighting* (SAW), which is also known as weighted linear combination or scoring method. In the SAW technique, the final score of each variable is computed as follows

2 $$\:{S}_{j}={\sum\:}_{i=1}^{n}{c}_{ij}{w}_{i}$$

Where $\:{S}_{j}$ is score for $\:{j}^{th}$ variable, and $\:{c}_{ij}$ is the normalised score for the $\:{j}^{th}$ variable, and $\:{w}_{i}$ is the weight of the $\:{j}^{th}$ variable. Then the final scores are ranked. This now implies that the higher the value of $\:{S}_{j}$ the higher is the rank. In addition to the above-described weights. We also used a well-known subjective method for determining weights in the analytical hierarchy process method (AHP) [16]. When applying the AHP, the importance of variables is determined using a pairwise comparison. For example, two variables of equal importance are assigned a value 1 and if a variable *j* is important than *k* then a value of 9 is assigned to variable *j*. The matrix of pairwise comparisons is created as $\:A=\left[{a}_{jk}\right]$. Where $\:{a}_{jk}=\frac{{I}_{j}}{{I}_{k}}$, $\:{I}_{j}$ and $\:{I}_{k}$ are the relative importance of variables j and k. We also have $\:{a}_{kj}=1/{a}_{jk}$ and $\:{a}_{jj}=1.$ In words, the elements of the pairwise comparison matrix, in the upper diagonal, will have values based on relative importance, the lower diagonal elements will be reciprocal of the upper diagonal elements and the diagonal elements will all be set to 1. Based on this matrix *A*, criteria weights were computed using “*ahpsurvey”* package [32]. Once the weights were computed using the AHP criteria we once again used Eq. 2 to create the overall score and rank the importance of a particular variable. All analyses were performed using R version 4.3.2, and the full code can be found https://github.com/sonianath/Super_Donor_Assesement_Tool.

This process was applied to participants who underwent a rigorous four-stage screening. Of the 208 initial participants, 93 individuals met the inclusion criteria. The Supplementary Table 2 summarises the characteristics of study participants across sociodemographic, lifestyle, behavioural, and clinical factors. The sample was predominantly female (61/93) and non‑Australian born (57/93). Mean daily sugar intake was 88.94 ± 41.86 g (low 56 ± 10.62 g, average 84.53 ± 8.02 g, high 138.80 ± 45.66 g) and mean fibre intake was 27.31 ± 14.05 g (low 18.58 ± 5.58 g vs. average 38.87 ± 13.55 g; *p* = 0.177) and 18 participants were smokers (19.4%). Oral‑health behaviours were generally favourable: 72/93 brushed twice or more daily, 25/93 flossed once or more per day, and 52/93 had a dental visit and topical fluoride treatment within the last 12 months. Forty participants had previous caries experience (4.32 ± 3.28 missing/filled teeth; *p* = 0.177). Mean stimulated saliva flow was 2.04 ± 0.73 mL/min (average 2.36 ± 0.59 vs. low 1.20 ± 0.18 mL/min), and saliva pH decreased from 7.57 ± 0.38 at baseline to 6.22 ± 0.84 after the glucose challenge (high 6.86 ± 0.42 vs. low 5.50 ± 0.58).

These final participants were evaluated with the SDAT, and their data were used to test three hypothetical donor models: *Optimal* (Model 1), *Ideal* (Model 2), and *Suboptimal donors* (Model 3), based on combinations of oral health, lifestyle, and diet history (Fig. 1B and C). The SDAT tool’s performance was validated by comparing microbiome profiles across these models, demonstrating the discriminatory power and reproducibility of the donor assessment framework.

### Microbial composition

**Fig. 2:**
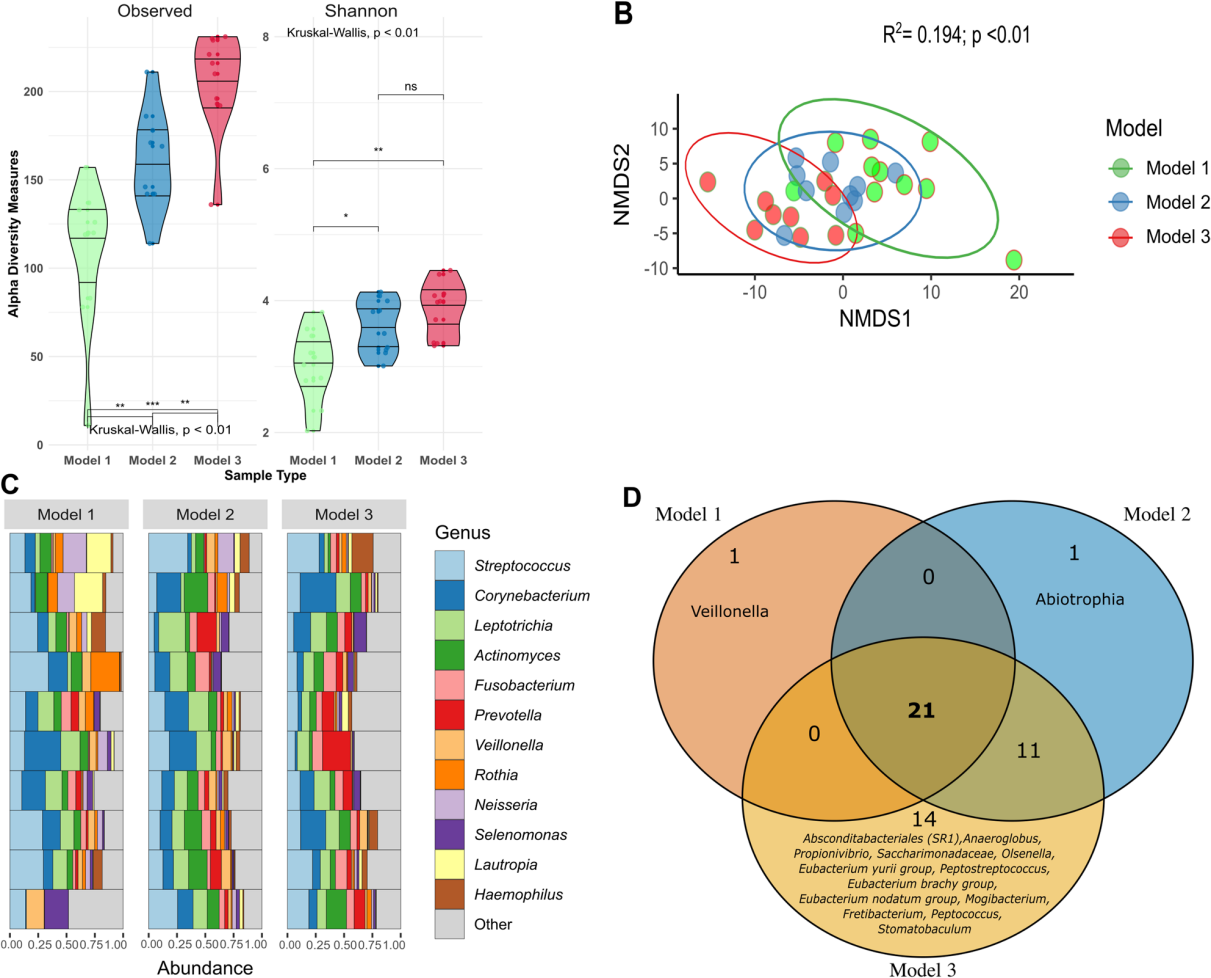
Comparison of Oral Microbiota Profiles Across Three Donor Selection Models. **A** Violin plots showing alpha diversity (Observed taxa and Shannon index) of plaque samples for each donor model: *“Model 1 Optimal donor”*, *“Model 2 Ideal donor”*, and “Model 3 *Suboptimal donor”.* Boxes indicate the interquartile range, with the median shown as a horizontal line. Statistical significance for alpha diversity was assessed using the Wilcoxon rank-sum test; **B** Non-metric multidimensional scaling (NMDS) ordination based on Aitchison distance, illustrating beta diversity and clustering of microbiota composition among the three donor models. Each point represents an individual sample, coloured by donor models. PERMANOVA showed a statistically significant difference among the models using *vegan* package. **C** Stacked bar plots depicting the relative abundance of the top 12 most prevalent microbial genera in plaque samples from each donor group. **D** Venn diagram illustrating the number of core genera (present in ≥ 80% of samples) unique to or shared among the three donor models

Observed richness for the *“Model 3 Suboptimal donors”* exhibited the highest diversity (Fig. 2A). The “*Model 2 ideal donors”* exhibited intermediate richness, whereas the *“Model 1 Optimal donor”* had the lowest richness and was significantly different from the other groups (*p* < 0.01). Pairwise comparisons revealed that both *“Ideal donors”* and *“Suboptimal donors”* had significantly higher richness than “*Optimal donors”* (p < 0.01 and p < 0.001, respectively), and that *“Suboptimal donors”* were also significantly higher than *“Ideal donors”* (p < 0.01). The Shannon diversity index similarly showed that the *“Suboptimal donors”* had the highest diversity, followed by Model 2 and then Model 1, with the difference significant (p < 0.01). Pairwise tests revealed that both Model 2 and Model 3 donors exhibited significantly higher Shannon diversity than Model 1 donors (p < 0.05 and p < 0.01), but the difference between Model 2 and Model 3 donors was not statistically significant. The NMDS plot showed partial separation among the three models (Fig. 2B). The permutational multivariate analysis of variance (PERMANOVA) revealed statistically significant differences in microbial community composition among the three donor models (F = 3.2576, R² = 0.1944, p < 0.01).

**Fig. 3:**
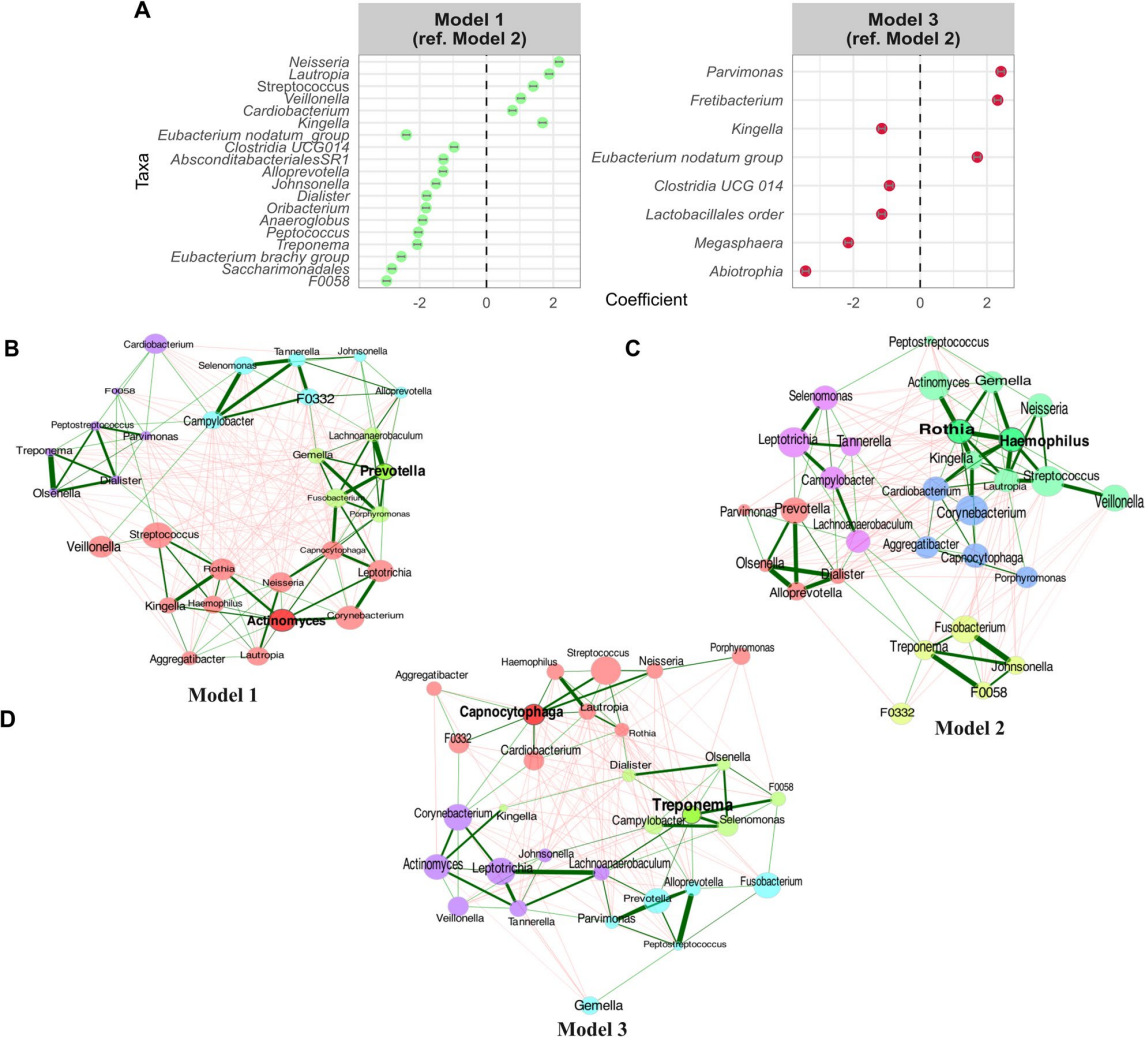
**A** Differential abundance analysis. **B-D** Network analysis of the three models (*Model 1 Optimal donor*, *Model 2 Ideal donor*, *Model 3 Suboptimal donor*) on a genus level with Pearson’s correlation with centre log-ratio transformation and zero handling using the *NetCoMi* package. The green edges indicate a positive association, and the red edges indicate a negative association. The nodes represent bacteria at the genus level, and the most important nodes (hubs) were highlighted by bold text and borders, and the node sizes were scaled using the Eigenvector centrality measure. The node colour represents the clusters network

For the relative abundance (Fig. 2C), all three models showed that the oral microbiome was dominated by *Streptococcus*, *Corynebacterium*, *Leptotrichia*, *Actinomyces*, *Fusobacterium*, *Prevotella*, *Veillonella*, *Rothia*, and *Neisseria*, among others. For core microbiome analysis, 21 genera were shared by all three models (Fig. 2D). The *“Optimal”* and *“Ideal donors”* had one unique core genus (*Veillonella*,* Abiotrophia*), and *“Suboptimal donors”* had 14 unique core genera, including *Absconditabacteriales*, *Anaeroglobus*, *Propionivibrio*, *Saccharimonadaceae*, and others. The greater number of unique core genera in *“Suboptimal donors”* suggests increased microbial diversity and complexity, likely reflecting the less favourable oral health and behavioural characteristics of this group. In contrast, the more streamlined core microbiomes of Models 1 and 2 are consistent with healthier, more stable oral environments. In the differential abundance analysis (Fig. 3A, Supplementary Table 3), six genera were significantly more abundant in Model 1 compared to Model 2 (used as reference), including *Neisseria*, *Lautropia*, *Streptococcus*, *Veillonella*, *Cardiobacterium*, and *Kingella*. While taxa *F0058*, *Saccharimonadales*,* Eubacterium brachy group*, *Treponema*, *Peptococcus*, *Anaeroglobus* were significantly lower in Model 1. Three genera, *Parvimonas*,* Fretibacterium*, and *Eubacterium nodatum group*, were more abundant in Model 3 compared to Model 2 (False Discovery Rate-adjusted *p* < 0.05).

In a network analysis of *“Optimal donors”* (Fig. 3B), *Actinomyces* forms a central hub, connected to several health-associated genera, including *Rothia*, *Streptococcus*, *Veillonella*, *Corynebacterium*,* Leptotrichia*, and *Lautropia*. This cluster also includes connections to *Kingella*, *Haemophilus*, and *Neisseria*, forming a core of health-associated bacteria. *Prevotella* serves as another central hub, connected to a distinct cluster of genera including *Gemella*, *Fusobacterium*, and *Porphyromonas*. Notably, *Prevotella* shows strong connections (thick edges) to both health-associated and potentially pathogenic genera, indicating its role as a bridge species. The network demonstrates significant bridge strength between the two major clusters through bridging taxa such as *Campylobacter*, and *F0332*. These connections suggest functional interdependence between the different microbial communities within the optimal donor oral microbiome. *For* Model 2 (Fig. 3C), *Rothia* and *Haemophilus* are the central hubs connected to genera including *Actinomyces*, *Gemella*, *Streptococcus*, *Veillonella*, *Kingella*, and *Lautropia*, and *Corynebacterium*. Network analysis of the oral microbiome in Model 3 (“Suboptimal donor”) identified *Treponema* and *Capnocytophaga* as the central hub genera within the microbial community. The *Treponema* cluster is associated with genera such as *Campylobacter* and *Selenomonas*, while the *Capnocytophaga* cluster is linked to *Cardiobacterium*, *Lautropia*,* Aggregatibacter*, *Streptococcus*, *Porphyromonas* and *Haemophilus*. The network structure for the *“Suboptimal donor”* (Fig. 3D) is characterised by a higher prevalence of genera commonly associated with periodontal disease and oral dysbiosis.

## Discussion

This study includes a comprehensive a priori screening of super donors using multicriteria evaluation, providing a robust assessment of donors and their oral microbiomes. The use of multi-criteria, evidence-informed donor selection tools developed in this study offers foundational safeguards and supports future clinical use of OMT. The SDAT provides a practical, evidence-based approach for identifying potential donors, particularly those from diverse backgrounds. The combination of weighted variables and candidate rankings provides a transparent framework for objective comparison across studies. The SDAT developed in this study was exploratory. The tool was constructed by defining and weighting a range of clinical, behavioural, and microbiological factors believed to influence oral health, and then grouping participants into three models: optimal, ideal, and suboptimal donors. This process involved generating and testing hypotheses about which combinations of factors best distinguish donor groups [33]. As such, the findings should be interpreted as hypothesis-generating and foundational for future confirmatory studies, rather than as definitive evidence of the assessment tool’s predictive validity.

This study objectively evaluates three models based on a four-stage screening process and a multicriteria decision-making process. The criteria for selecting super donors were diverse and multifaceted, and each criterion was weighted differently to prioritise specific attributes. As a result, different donors excelled in different aspects of the selection criteria. However, given the complexity and diversity of individual oral microbiomes and lifestyle factors, having a single “super” donor would be unrealistic, and no donor ranked highest across all 12 dimensions. The flexibility of the super donor assessment captures various aspects of oral health and lifestyle factors, and the tool can be tailored to specific goals and desired outcomes to select a super donor for a personalised oral health intervention and is the foundation to trial OMT for dental caries safely. While the current model was tailored for donor selection in caries prevention, the framework is readily adaptable to other oral diseases, including periodontitis. The model’s design promotes transparency, replicability, and adaptability for different disease targets. The super donor assessment criteria were derived from a broad literature review and based on known risk and protective factors in oral health epidemiology, based on lifestyle and dental behavioural factors [6, 9]. These bacterial species were selected because they are well-established markers for oral disease states; *S. mutans* is the principal etiological agent of dental caries due to its acidogenic and biofilm-forming capabilities [34]; *P. gingivalis*, *T. denticola*, and *T. forsythia* form the “red complex”, and *F. nucleatum* is strongly associated with periodontal disease progression and host tissue destruction [35, 36]. Including both cariogenic and periodontitis-linked species ensures a comprehensive assessment of oral health risk profiles in the model. Although advancements in the understanding of oral ecology consistently challenge the notion of fixed ‘key pathogens.’ Increasingly, oral disease is recognised as a result of community-level dysbiosis rather than the mere presence of individual pathogenic species. Ecological approaches emphasise the interplay and balance among multiple microbial residents, including bacteria, fungi, viruses, and others, where shifts in network dynamics can convert commensals into disease-promoting taxa [37]. Thus, as ecological concepts and analytic technologies progress, revision of currently accepted ‘key pathogens’ is likely, with a growing emphasis on community structure, functional resilience, and ecological interactions in risk assessment and donor selection.

The microbial composition analysis among the three donor models showed a PERMANOVA R² of 0.194, indicating donor selection explained about 19.4% of the variance in oral microbiome structure. However, the remaining unexplained variance (approximately 80%) reflects the influence of unmeasured host factors (e.g., genetics, immune function), temporal fluctuations in the microbiome, and stochastic ecological processes that were not incorporated into the current model. Model 1 demonstrates several characteristics that support its designation as a potential super donor, despite exhibiting the lowest alpha diversity. The differential abundance analysis revealed health-associated genera, including *Neisseria*, *Lautropia*, *Streptococcus*, and *Veillonella*, which are typically associated with oral health maintenance and caries prevention [38]. Network analysis identified *Actinomyces* and *Prevotella* as central hubs, suggesting a functionally organised microbial community in which even potentially pathogenic genera, such as *Prevotella*, are maintained in beneficial configurations through network-level regulatory mechanisms. This supports the theoretical framework that prioritises clinical outcomes over lifestyle factors, suggesting that Model 1 donors possess resilient, health-promoting microbiomes despite potentially unfavourable environmental factors. Model 2 represents the most balanced profile, with intermediate alpha diversity and network analysis revealing *Rothia* and *Haemophilus* as central hubs. The presence of these taxa suggests optimal functional organisation combining specialised metabolic functions with cross-habitat microbial communication. Model 2 represents the theoretical ideal where excellent oral health synergises with favourable lifestyle habits to produce highly organised, stable microbial networks. Few individuals may naturally have more resilient oral microbiota than others, providing them with a natural defence system against dental caries and periodontal disease [39]. The presence of health-associated bacteria can have a protective mechanism against caries formation [39]. These commensals form a protective biofilm on the tooth surface, which acts as a physical barrier and helps maintain a balanced microbial community. While cariogenic pathobionts are often already present within the biofilm, the dominance of commensals can limit their overgrowth by producing substances that neutralise acids, an effect influenced largely by the individual’s salivary flow rate. Additionally, commensals may exert anti-inflammatory effects and compete with cariogenic species for nutrients, collectively supporting oral health [40, 41]. The presence of health-associated bacteria can tilt the balance and halt the progression of dental caries [40]. Therefore, when selecting a super donor for OMT individuals from Models 1 and 2 could be prioritised as they have a higher likelihood of successful oral health outcomes.

Model 3 exhibited the highest alpha diversity but displayed concerning network characteristics, with *Treponema* and *Capnocytophaga* as central hubs and both genera strongly associated with periodontal disease and oral dysbiosis [35]. While high diversity might superficially appear beneficial, in this context it likely reflects an unstable, pathogen-dominated ecosystem. The differential abundance analysis showed significant depletion of beneficial genera, including *Parvimonas*,* Kingella*, and *Megasphaera*, compared to Ideal donors, further supporting the dysbiotic nature of these communities [36, 42, 43]. Since all the participants were disease-free, the health-associated microbiota may have driven the change. Additionally, harbouring a pathogenic genus could increase the risk of future oral conditions, leading to a shift in the ecological balance. High sugar consumption promotes the proliferation of acidogenic and aciduric bacteria, leading to dental caries [44].

A few limitations were present in this study: (1) The inclusion of the abundance of oral pathogens as one of its dimensions in the SDAT. This introduces a risk that subsequent analyses comparing the microbiome profiles of the groups may be biased, as information about the outcome (microbiota composition) was already used in the process of group formation [45]; (2) Sequencing of the V4 hypervariable region of the bacterial 16S rRNA gene, as this approach offers lower taxonomic resolution than full-length 16S rRNA sequencing. In the context of our study, which focused on genus-level relative abundance and the identification of five key pathogenic species (*S. mutans*,* F. nucleatum*, *P. gingivalis*, *T. denticola*, *T. forsythia*), the V4 region provided adequate resolution for the primary objectives. However, we acknowledge that truncating to five pathobiont species and performing genus-level analysis may have excluded additional key taxa that could contribute to donor microbiome profiles; (3) various host and environmental factors influence the oral microbiome, and other unmeasured variables may still have an effect; (4) There may also be bias in participant selection, as this study relied on volunteers; (5) A formal sensitivity analysis was not performed and this limits the ability to assess the robustness of our models to outliers or analytical decisions.

In addition to bacteria, the oral microbiome comprises diverse non-bacterial constituents, mainly fungi (e.g., *Candida spp*.), viruses (*bacteriophages* and *herpesviruses*), archaea, and other eukaryotes [37]. These non-bacterial taxa may act as latent pathogens or ecological modulators, influencing oral health directly through their pathogenic potential or indirectly by shaping bacterial community structure and immune responses. OMT strategies in the future and super donor definitions should therefore consider the broader ecological context, incorporating non-bacterial elements to support optimal oral health outcomes. Future research should also integrate multi-omics approaches, metagenomics, metabolomics, and proteomics to more comprehensively characterise super donors and improve the precision of donor-recipient matching. These multi-omics data could be incorporated into the SDAT framework using the same multicriteria decision-making methodology, with additional weighted dimensions reflecting functional and strain-level characteristics. Additionally, an immune screening of recipient participants with the lowest induction of pro-inflammatory cells could be done to help prevent OMT rejection. Such an integrated approach would enhance both the biological validity and clinical utility of super donor assessment for personalised oral microbiome transplantation. Also, confirmatory studies should employ longitudinal designs, consider full-length 16S rRNA sequencing or shotgun metagenomics to capture strain-level diversity and functional genetic potential, expanded covariate sets, and machine learning approaches to improve model fit and predictive capacity for clinical outcomes following OMT. While the present study focused on a caries prevention model, the SDAT framework and donor selection process are readily adaptable to other oral disease contexts, including periodontitis. The differential network analysis and pathobiont abundance screening directly address periodontal risk markers. Future research should define disease-specific donor selection criteria and validate OMT for periodontitis through targeted trials.

Several additional factors should be considered when developing novel microbiota donor pools. (1) Donor pool: creating a pool of diverse super donors. It would be pragmatic, as no single donor could fulfil all the criteria, given the wide range of participants who excel in different criteria. By establishing a donor pool, each donor’s unique microbiome may contribute to different beneficial characteristics to the recipient’s oral environment; (2) Multiple criteria evaluation, which includes a combination of sociodemographic, lifestyle and oral health factors for donor selection; (3) Customised donor selection, for a personalised and targeted OMT. The donor selection should be customisable based on recipient needs, e.g., transplants for dental caries would differ from that of periodontal disease; (4) Recipient selection, a cross-matching should be carried out based on socio-demographics, lifestyle, and oral health factors should be carried out between donor and recipient; (5) The establishment of a biobank for donor samples can reduce the need for continuous donor monitoring, as stored samples can be thoroughly screened and qualified at the time of collection.

## Conclusion

This study highlights the comprehensive evaluation of super donor criteria using a standardised multicriteria decision-making process and the potential to identify donors for personalised transplantation and reduced adverse events. Multiple models allow a broader pool of potential super donors with specific strengths in different areas. The approach offers enhanced safeguards for donor selection and provides a foundation specifically for testing OMT as a preventive strategy in dental caries.

## Supplementary Information

Supplementary Material 1.

## Data Availability

All sequence data generated in this project are available at NCBI under BioProject ID PRJNA1311717. Clinical trial number: not applicable. The R scripts to analyse the 16 S rRNA amplicon sequencing data are available at https://github.com/sonianath/Super_Donor_Assesement_Tool.

## Abbreviations

AHP: Analytical hierarchy process
ASV: Amplicon sequence variant
DMFT: Decayed, missing, filled tooth
OMT: Oral microbiome transplantation
LCA: Latent class analysis
MCDM: Multiple criteria decision-making
NMDS: Non-metric multidimensional scaling
PERMANOVA: Permutational multivariate analysis of variance
SDAT: Super donor assessment tool
