# Supplementary material for: Super donor assessment tool for oral microbiome transplantation
Source: BMC Microbiol. 2025 Dec 23;26:56. doi: 10.1186/s12866-025-04630-z (PMC12836760; doi:10.1186/s12866-025-04630-z)
Supplement: Supplementary file 1 — Supplementary Material 1. [file 12866_2025_4630_MOESM1_ESM.docx]

Title: Super Donor Assessment Tool for Oral Microbiome Transplantation

Supplementary Material

Table of Contents

[1. Supplementary text 1 2](#_Toc214963108)

[1.1 Covariate categorisation and sample collection 2](#_Toc214963109)

[1.1.1 Covariates 2](#_Toc214963110)

[1.1.2 Sample collection 2](#_Toc214963111)

[1.2 Selection of covariates and weight distribution for the super donor assessment tool 3](#_Toc214963112)

[Selection of covariates: 3](#_Toc214963113)

[Weigh calculation: 3](#_Toc214963114)

[2. Supplementary text 2 Data preprocessing 4](#_Toc214963115)

[3. Supplementary Tables 5](#_Toc214963116)

[3.1 Supplementary Table 1 Ranking and weight distribution for the covariates for super donor selection models 5](#_Toc214963117)

[3.2 Supplementary Table 2. The socio-demographic, lifestyle and oral health behavioural data of the study participants (N=93) 7](#_Toc214963118)

[3.3 Supplementary Table 3. Differential abundance analysis at the genus level, for the three models, controlling for age and sex, and using Model 2 as the reference group. 9](#_Toc214963119)

# Supplementary text 1

## Covariate categorisation and sample collection

### Covariates

*Lifestyle factors:* (i) Physical activity was captured using a short form of the International Physical Activity Questionnaire (IPAQ-SF) [1]. The IPAQ-SF consists of three domains with seven questions: vigorous physical activity (VPA), moderate physical activity (MPA), and walking and sitting evaluated for the past week. Within each item, the number of days and the time spent were recorded. The weekly physical activity by a metabolic equivalent (MET-min/week) was calculated by multiplying the times spent in each domain per week by a pre-determined weighing factor (Walking: 3.3 METs, MPA: 4.0 METs, VPA: 8.0 METs) [1]. Then, the scores were summed across domains. Per the guideline, activity less than 10 minutes was considered 0, and all activity was truncated to 3 hours. The participants were categorised into three groups: minimal activity (0- 600 MET min/week), moderate activity (601- 1500 MET min/week), and high activity (<600 MET min/week). (ii) Smoking was defined as anyone currently smoking.

*Dietary factors:* (i) Diet: High sugar consumption was defined as more than 101 gm/day, average as >71 gm/day and <100 gm/day, and low as <70 gm/day. (ii) According to Australian standards, low fibre consumption was defined as less than 24 gm/day for females and 30 gm/day for males.

*Oral health behaviours* measured were tooth brushing frequency (> two times/day vs < once daily), last dental visit (<12 months vs. >12 months), and lifetime exposure to topical fluoride treatment as ‘yes’ and ‘no’ answer.

### Sample collection

The dental plaque and saliva collections were performed before the oral assessments to minimise the influence of probing. On the day of sample collection, participants were advised to abstain from brushing, using mouthwash, eating, or drinking for at least one hour prior. Six sites were selected for a pooled plaque collection using a sterile dental curette. The plaque was collected in an Eppendorf tube containing 200 μL of sterile phosphate-buffered solution (PBS) for downstream processing.

The whole stimulated saliva was collected by chewing sugar-free chewing gum for 2 min and then collected for 5 min. The saliva flow rate was measured, and the expectorated saliva was collected in a tube for glucose challenge tests [2]. The saliva collected at baseline pH was recorded and categorised as high if higher than 7.3, average between 6.8-7.3, and <6.7 as low. The saliva flow rate was “average” for a flow rate of ≥1.6 ml/min and low for 1.5 ml/min or less.

Glucose challenge test: The saliva was subjected to a glucose test by adding 500 µL of 20% glucose to each 5 ml saliva sample, and the pH was reassessed after 2 hours using the pH titrator (916 Ti-Touch, Metrohm). The saliva pH was sub-grouped into low if the pH was lower than 6.33 and high if above.

## Selection of covariates and weight distribution for the super donor assessment tool

Selection of covariates: A multicriteria decision-making approach (MCDM) was used to select the variables [3]. The first step was to identify the key criteria for selecting super donors. Based on the literature, 12 dimensions were used, including dental health markers, nutritional, lifestyle, behavioural, and microbial factors [4, 5]. Dental health markers included the final pH difference after the glucose test, salivary flow rate, the number of missing teeth, the number of restored or filled teeth and fluoride application, specifically whether a fluoride varnish had been applied. Sugar consumption and dietary fibre consumption were assessed to evaluate nutritional habits. Smoking status, the timing of the last dental visit for treatment, brushing frequency, and physical activity were included to capture lifestyle factors influencing oral health. Finally, the bacterial composition of the oral microbiome was evaluated by quantifying the abundance of key species: *Streptococcus mutans* (*S. mutans*), *Fusobacterium nucleatum* (*F. nucleatum*), *Porphyromonas gingivalis* (*P. gingivalis*), *Treponema denticola* (*T. denticola*), and *Tannerella forsythia* (*T. forsythia*).

Weigh calculation: Weights for each donor selection criterion were determined using analytical hierarchical process (AHP), incorporating pairwise comparisons across four domains: lifestyle factors, oral health behavioural factors, clinical factors, and microbial factors. Each of these factors was assigned a specific weight in the assessment tool to reflect its relative importance in defining a super donor. Within the lifestyle category, sugar consumption (W = 0.158) and smoking (W = 0.086) received the highest weights, while physical activity (W = 0.004) and fibre consumption (W = 0.038) received lower weights. Oral health behavioural factors, including last dental visit (W = 0.022), tooth brushing frequency (W = 0.025), and fluoride application or use (W = 0.023), were each assigned modest weights. Clinical factors such as restored or filled teeth (W = 0.063), missing teeth (W = 0.069), stimulated saliva flow rate (W = 0.053), and final pH difference after glucose (W = 0.08) were also incorporated, reflecting their relevance to oral health status. Microbial factors were given substantial emphasis, with *S. mutans* (W = 0.122), *P. gingivalis* (W = 0.092), *T. forsythia* (W = 0.087), *T. denticola* (W = 0.047), and *F. nucleatum* (W = 0.030) included as key indicators of oral microbial risk. The resulting weights were used to generate a composite donor suitability score, and the top 10 super donor candidates were selected using each model (Supplementary Table 1).

# Supplementary text 2 Data preprocessing

QIIME version software (v.2022.11) [6] was utilised for processing and filtering high-quality reads “*qiime demux summarize*” function. The filter reads were demultiplexed within QIIME2 using *q2-deblur* plugin. Samples containing less than 100 DNA sequences were excluded from further analysis. The reads were trimmed to 230 and 210 for forward and reverse reads. Representative sequences were generated using the “*q2-feature-table-tabulate*-seqs” command. The “*qiime phylogeny fasttree*” command was used to construct a phylogenetic tree. Next, the Amplicon Sequence Variants (ASVs) were created, and taxonomies were assigned using the Silva 138-138-99-nb classifier database. The initial exploration of data was done using “*qiime diversity core-metrics-phylogenetic*” function to observe the differences in the test and control samples using beta diversity plots. In a PCoA plot using Bray-[5]Curtis distances, there was a clear separation of test and control samples (PERMANOVA p<0.01). As a result, R Studio version 2022.02.2. was used to remove potential contaminants from the samples. The packages *decontam* [7], *phyloseq* [8] and *qiime2R* [9] were used to detect and remove microbes from three types of negative controls (extraction blank control, no template control, and curette wash) using a two-step process. The method employed is described previously [4, 5].

# Supplementary Tables

## Supplementary Table 1 Ranking and weight distribution for the covariates for super donor selection models

| **Factors** | **Description** | **Model 1 Optimal donor** | **Model 2 Ideal donor** | **Model 3 Sub-optimal donor** |
| --- | --- | --- | --- | --- |
| **Lifestyle factors** | Smoking  W= 0.086 | 1. No 2. Yes | 1. No 2. Yes | 1. Yes 2. No |
|  | Physical activity  W= 0.004 | 1. No /less exercise. 2. ≧ 75 min/week | 1. ≧ 75 min/week 2. No /less exercise | 1. No /less exercise 2. ≧ 75 min/week |
|  | Sugar consumption  W= 0.158 | 1. ≧ 100 2. ≧ 70 ≦ 99 3. < 69 | 1. < 69 2. ≧ 70 ≦ 99 3. ≧ 100 | 1. ≧100 2. ≧ 70 ≦ 99 3. < 69 |
|  | Fibre consumption  W= 0.038 | 1. ≦ 20 2. ≧ 21 | 1. ≧ 21 2. ≦ 20 | 1. ≦ 20 2. ≧ 21 |
| **Oral health behavioral** | Last dental visit  W= 0.022 | 1. ≧ 12 months 2. <12 months | 1. < 12 months 2. ≧ 12 months | 1. ≧ 12 months 2. < 12 months |
|  | Tooth Brushing Frequency  W= 0.025 | 1. Once 2. Twice | 1. Twice 2. Once | 1. Once 2. Twice |
|  | Fluoride application/use  W= 0.023 | 1. No 2. Yes | 1. Yes 2. No | 1. No 2. Yes |
| **Clinical Factors** | Restored/Filled teeth  W= 0.063 | 1. 0 2. 1-3 3. ≧ 4 | 1. 0 2. 1-3 3. ≧ 4 | 1. ≧ 4 2. 1-3 3. 0 |
|  | Missing teeth  W= 0.069 | 1. 0 2. 1-3 3. ≧ 4 | 1. 0 2. 1-3 3. ≧ 4 | 1. ≧ 4 2. 1-3 3. 0 |
|  | Stimulated saliva flow rate  W= 0.053 | 1. 1.5-2.0 2. ≧ 2.1 3. ≦ 1.4 | 1. 1.5-2.0 2. ≧ 2.1 3. ≦ 1.4 | 1. ≦ 1.4 2. ≧ 2.1 3. 1.5-2.0 |
|  | Final pH difference after the glucose test  W= 0.08 | 1. ≦ 0 – 0.65 2. 0.66 – 1.5 3. ≧ 1.6 | 1. ≦ 0 – 0.65 2. 0.66 – 1.5 3. ≧ 2 | 1. ≧ 1.6 2. 0.66 – 1.5 3. ≦ 0 – 0.65 |
| **Microbial Factors** | *Streptococcus mutans*  W = 0.122 | 1. 0 abundance 2. ≧ 0.0001 | 1. 0 abundance 2. 0.0001-0.01 | 1. ≧ 0.0001 2. 0 abundance |
|  | *Porphyromonas gingivalis*  W= 0.092 | 1. 0 abundance 2. ≧ 0.0001 | 1. 0 abundance 2. ≧ 0.0001 | 1. ≧ 0.0001 2. 0 abundance |
|  | *Tannerella Forsythia*  W= 0.087 | 1. 0 abundance 2. ≧ 0.0001 | 1. 0 abundance 2. ≧ 0.0001 | 1. ≧ 0.0001 2. 0 abundance |
|  | *Treponema denticola*  W= 0.047 | 1. 0 abundance 2. ≧ 0.0001 | 1. 0 abundance 2. ≧ 0.0001 | 1. ≧ 0.0001 2. 0 abundance |
|  | *Fusobacterium nucleatum*,  W= 0.030 | 1. 0 abundance 2. ≧ 0.0001 | 1. 0 abundance 2. ≧ 0.0001 | 1. ≧ 0.0001 2. 0 abundance |

## Supplementary Table 2. The socio-demographic, lifestyle and oral health behavioural data of the study participants (N=93)

| **Factors** | **Category** | **Subgroups** | **N** | **Mean ± SD** | ***p-value*** |
| --- | --- | --- | --- | --- | --- |
| **Sociodemographic** | Age | All | 93 | 31.68 ± 11.73 | 0.46 |
|  |  | Young adults | 50 | 24.30 ± 2.41 |  |
|  |  | Adults | 43 | 40.25 ± 12.42 |  |
|  | Gender | Male | 32 |  | 0.002 |
|  |  | Female | 61 |  |  |
|  | Country of birth | Australian | 36 |  | 0.029 |
|  |  | Other | 57 |  |  |
|  | Level of education | Tertiary education | 57 |  | 0.002 |
|  |  | Secondary education or less | 36 |  |  |
|  | Employment | Employed | 61 |  | 0.002 |
|  |  | Not working | 32 |  |  |
| **Lifestyle** | Physical activity | Minimal activity (<600 met min) | 35 |  | 0.405 |
|  |  | Moderate activity (600-1500 met min) | 25 |  |  |
|  |  | High activity (> 1500 met min) | 33 |  |  |
|  | Daily sugar intake (gm/d) | Overall | 93 | 88.94 ± 41.86 | 0.507 |
|  |  | Low | 35 | 56 ± 10.62 |  |
|  |  | Average | 32 | 84.53 ± 8.02 |  |
|  |  | High | 26 | 138.80 ± 45.66 |  |
|  | Daily fibre intake (gm/d) | Overall | 93 | 27.31 ± 14.05 | 0.177 |
|  |  | Low (<24 gm/day) | 53 | 18.58 ± 5.58 |  |
|  |  | Average (25 -30 gm/d) | 40 | 38.87 ± 13.55 |  |
|  | Smoking | Smoker | 18 |  | <0.0001* |
|  |  | Non-smoker | 75 |  |  |
| **Clinical Factors** | Tooth brushing frequency | Once | 21 |  | <0.0001* |
|  |  | Twice or more | 72 |  |  |
|  | Flossing frequency | Once or more everyday | 25 |  | <0.0001* |
|  |  | Occasionally/never | 68 |  |  |
|  | Last dental visit | Less than 12 months | 52 |  | 0.254 |
|  |  | More than 12 months | 41 |  |  |
|  | Topical fluoride treatment | Yes | 52 |  | 0.254 |
|  |  | No | 41 |  |  |
|  | Caries experience | Missing and filled teeth | 40 | 4.32 ± 3.28 | 0.177 |
|  |  | No caries experience | 53 |  |  |
|  | Saliva flow rate | Overall | 93 | 2.04± 0.73 | <0.0001* |
|  |  | Average | 68 | 2.36± 0.59 |  |
|  |  | Low | 25 | 1.20 ± 0.18 |  |
|  | Saliva pH baseline | Overall | 93 | 7.57 ± 0.38 | 0.604 |
|  | Saliva pH post-glucose test | Overall | 93 | 6.22 ± 0.84 |  |
|  |  | High | 49 | 6.86 ± 0.42 |  |
|  |  | Low | 44 | 5.50 ± 0.58 |  |

Independent *t-test* for intragroup comparison for continuous variables using mean and S. For categorical values Chi square test was calculated, and ANOVA for multiple group comparisons.

*The p-value was <0.05 was considered significant.

## Supplementary Table 3. Differential abundance analysis at the genus level, for the three models, controlling for age and sex, and using Model 2 as the reference group.

| **Feature** | **Value** | **Coefficient** | **Standard error** | ***p-value*** |
| --- | --- | --- | --- | --- |
| *F0058* | Model 1 | -2.99 | 0.57 | <0.01 |
| *Neisseria* | Model 1 | 2.17 | 0.55 | <0.01 |
| *Treponema* | Model 1 | -2.07 | 0.59 | <0.01 |
| *Saccharimonadales* | Model 1 | -2.83 | 0.84 | <0.01 |
| *Oribacterium* | Model 1 | -1.81 | 0.54 | <0.01 |
| *Eubacterium nodatum group* | Model 1 | -2.40 | 0.74 | <0.01 |
| *Dialister* | Model 1 | -1.79 | 0.57 | <0.01 |
| *Streptococcus* | Model 1 | 1.40 | 0.45 | <0.01 |
| *Eubacterium brachy group* | Model 1 | -2.55 | 0.85 | <0.01 |
| *Peptococcus* | Model 1 | -2.04 | 0.69 | <0.01 |
| *Kingella* | Model 1 | 1.68 | 0.58 | <0.01 |
| *Lautropia* | Model 1 | 1.88 | 0.65 | <0.01 |
| *Alloprevotella* | Model 1 | -1.30 | 0.49 | 0.01 |
| *Johnsonella* | Model 1 | -1.51 | 0.58 | 0.01 |
| *Absconditabacteriales SR1* | Model 1 | -1.29 | 0.58 | 0.03 |
| *Clostridia UCG 014* | Model 1 | -0.97 | 0.47 | 0.04 |
| *Veillonella* | Model 1 | 1.03 | 0.51 | 0.04 |
| *Cardiobacterium* | Model 1 | 0.78 | 0.39 | 0.05 |
| *Anaeroglobus* | Model 1 | -1.91 | 0.96 | 0.05 |
| *Megasphaera* | Model 3 | -2.15 | 0.17 | <0.01 |
| *Parvimonas* | Model 3 | 2.42 | 0.47 | <0.01 |
| *Abiotrophia* | Model 3 | -3.43 | 0.68 | <0.01 |
| *Fretibacterium* | Model 3 | 2.32 | 0.50 | <0.01 |
| *Eubacterium nodatum group* | Model 3 | 1.71 | 0.57 | <0.01 |
| *Lactobacillales order* | Model 3 | -1.15 | 0.53 | 0.03 |
| *Kingella* | Model 3 | -1.15 | 0.58 | 0.05 |
| *Clostridia UCG 014* | Model 3 | -0.92 | 0.47 | 0.05 |

Reference

1. Craig CL, Marshall AL, Sjöström M, Bauman AE, Booth ML, Ainsworth BE, et al. International physical activity questionnaire: 12-country reliability and validity. Medicine & science in sports & exercise. 2003;35(8):1381-95.

2. Nath S, Zilm P, Jamieson L, Kapellas K, Goswami N, Ketagoda K, et al. Development and characterization of an oral microbiome transplant among Australians for the treatment of dental caries and periodontal disease: A study protocol. PLoS One. 2021;16(11):e0260433.

3. Roszkowska E. Rank ordering criteria weighting methods–a comparative overview. Optimum Studia Ekonomiczne. 2013;(5 (65)):14-33.

4. Nath S, Zilm P, Jamieson L, Ketagoda DHK, Kapellas K, Weyrich L. Characterising healthy Australian oral microbiomes for 'super donor' selection. J Dent. 2024;151:105435; doi: 10.1016/j.jdent.2024.105435.

5. Nath S, Zilm P, Jamieson L, Santiago PHR, Ketagoda DHK, Weyrich L. The influence of diet, saliva, and dental history on the oral microbiome in healthy, caries-free Australian adults. Scientific Reports. 2025;15(1):18755; doi: 10.1038/s41598-025-03455-0.

6. Bolyen E, Rideout JR, Dillon MR, Bokulich NA, Abnet CC, Al-Ghalith GA, et al. Reproducible, interactive, scalable and extensible microbiome data science using QIIME 2. Nature Biotechnology. 2019;37(8):852-7; doi: 10.1038/s41587-019-0209-9.

7. Davis NM, Proctor DM, Holmes SP, Relman DA, Callahan BJ. Simple statistical identification and removal of contaminant sequences in marker-gene and metagenomics data. Microbiome. 2018;6(1):226; doi: 10.1186/s40168-018-0605-2.

8. McMurdie PJ, Holmes S. phyloseq: an R package for reproducible interactive analysis and graphics of microbiome census data. PLoS One. 2013;8(4):e61217; doi: 10.1371/journal.pone.0061217.

9. Bisanz JE. qiime2R: Importing QIIME2 artifacts and associated data into R sessions. Version 099. 2018;13.
